# Comparative activity of ceftobiprole against coagulase-negative staphylococci from the BSAC Bacteraemia Surveillance Programme, 2013–2015

**DOI:** 10.1007/s10096-018-3295-6

**Published:** 2018-06-06

**Authors:** Anne Santerre Henriksen, Jennifer Smart, Kamal Hamed

**Affiliations:** Basilea Pharmaceutica International Ltd., Grenzacherstrasse 487, 4005 Basel, Switzerland

**Keywords:** Antimicrobial resistance, Bacteraemia Surveillance Programme, British Society for Antimicrobial Chemotherapy, Ceftobiprole, Coagulase-negative staphylococci, Susceptibility testing

## Abstract

Coagulase-negative staphylococci (CoNS) are a significant cause of bacteraemia, the treatment of which is becoming increasingly complex due to the emergence of multidrug-resistant strains. This study aimed to evaluate the in vitro activity of ceftobiprole, an advanced-generation cephalosporin, as compared with other antimicrobial agents against CoNS from patients with bacteraemia. As part of the British Society for Antimicrobial Chemotherapy (BSAC) Bacteraemia Surveillance Programme, 650 blood isolates of CoNS were obtained from patients with bacteraemia at 74 centres throughout the UK and Ireland for the years 2013–2015. Minimum inhibitory concentrations (MICs) of ceftobiprole and other antimicrobial agents were determined using the BSAC agar dilution method. Susceptibility was assessed by European Committee on Antimicrobial Susceptibility Testing (EUCAST) criteria. The majority of the isolates (63.2%) were *Staphylococcus epidermidis*. Overall, methicillin resistance, as determined by oxacillin susceptibility testing, was observed in 64.2% of isolates. The MIC_50/90_ of ceftobiprole was 1/2 mg/L, and 100% of CoNS isolates were inhibited at the EUCAST ceftobiprole non-species-specific pharmacokinetic/pharmacodynamic breakpoint of 4 mg/L. Only one isolate was resistant to vancomycin. Overall rates of resistance to ciprofloxacin, clindamycin, erythromycin and teicoplanin were 50.5, 25.1, 68.2 and 20.9%, respectively. In *S. epidermidis*, resistance to oxacillin was associated with increased resistance to other antimicrobials. Ceftobiprole demonstrated in vitro activity against all CoNS species isolated from patients with bacteraemia and was active against species resistant to other antistaphylococcal antimicrobials. The collection of clinical data regarding the efficacy of ceftobiprole in treating CoNS bacteraemia is warranted.

## Introduction

Coagulase-negative staphylococci (CoNS) are ubiquitous colonisers of human skin and mucous membranes that have the potential to cause clinically significant infections [1]. CoNS are a major cause of nosocomial infection, with a particular risk in premature newborns, patients with neutropenia and patients undergoing invasive procedures [1–3]. Due in part to their affinity for synthetic substrates and ability to form biofilms, CoNS are a frequent cause of device-related local and systemic infections [1]. These include catheter-related bloodstream infections (CRBSIs) and infections of implants, such as prosthetic heart valves and orthopaedic devices [1]. Device-related infections are particularly difficult to treat, and removal of the device may be required [1, 4]. Developments in healthcare, including higher proportions of elderly and comorbid patients and more frequent use of medical/surgical procedures involving insertion or implantation of synthetic devices, have increased the rate of infections with CoNS [1, 4]. The incidence of resistance to methicillin (as determined by oxacillin susceptibility testing), as well as to other classes of antimicrobial agents, is high among CoNS [1]. Emergence of these multidrug-resistant strains has further complicated the treatment of CoNS infections.

Ceftobiprole is an advanced-generation, broad-spectrum cephalosporin that binds to several penicillin-binding proteins (PBPs), including PBP2a, and is therefore active against methicillin-resistant (MR) staphylococci [5]. Ceftobiprole medocaril, the prodrug form of ceftobiprole, has been approved in several European and non-European countries for the treatment of hospital-acquired pneumonia, excluding ventilator-associated pneumonia, and for community-acquired pneumonia caused by susceptible Gram-positive and Gram-negative pathogens including MR *Staphylococcus aureus* [6, 7]. Ceftobiprole has demonstrated in vitro potency and bactericidal activity against methicillin-susceptible (MS) and MR strains of *S. aureus*, as well as against CoNS [8]. This study was conducted in order to determine the activity of ceftobiprole against a total of 650 isolates of several different CoNS species collected between 2013 and 2015 from patients with bacteraemia in the UK and Ireland.

## Materials and methods

### Bacterial isolates

A total of 74 centres from across the UK and Ireland participated in the British Society for Antimicrobial Chemotherapy (BSAC) Bacteraemia Surveillance Programme. Up to seven CoNS isolates per centre per year were obtained from the blood of patients with clinically significant bacteraemia. Repeat isolates from the same clinical episode were excluded. The isolates were sent to the central testing laboratory (Public Health London, Colindale, London), along with relevant patient demographic information. At the central testing laboratory, the isolates were identified to the species level using the matrix-assisted laser desorption ionisation-time of flight (MALDI-TOF) mass spectrometry. For the surveillance year 2013, the isolates were identified by MALDI-TOF mass spectrometry in conjunction with ChromAgar (Becton Dickinson) and the coagulase tube test.

### Antimicrobial susceptibility testing

Minimum inhibitory concentrations (MICs) of ceftobiprole and comparators were determined by the BSAC agar dilution method [9, 10]. Briefly, when testing staphylococci, 10^4^ colony-forming units per spot are applied to Iso-Sensitest agar containing doubling dilutions of antimicrobial agents, except for oxacillin where Columbia agar plus 2% NaCl is used. Determination of methicillin resistance was based on the European Committee on Antimicrobial Susceptibility Testing (EUCAST) oxacillin breakpoints: > 2 mg/L for *Staphylococcus lugdunensis* and *Staphylococcus saprophyticus* and > 0.25 mg/L for other CoNS species [11]. As there are currently no species-specific breakpoints for ceftobiprole against CoNS, the ceftobiprole non-species-specific pharmacokinetic/pharmacodynamic (PK/PD) breakpoint of 4 mg/L was used to determine ceftobiprole resistance [7, 11]. Resistance to the other antimicrobial agents (ciprofloxacin, clindamycin, erythromycin, teicoplanin and vancomycin) was determined based on EUCAST breakpoints [11]. MIC_50_ and MIC_90_ were determined for the entire collection of isolates and for individual species with at least ten isolates collected.

#### Data availability

The datasets generated and/or analysed during the current study are available from the corresponding author on reasonable request.

## Results

A total of 650 CoNS isolates were collected, with consistent numbers obtained in each year of the study. The isolates were collected from inpatients and outpatients at both university and community hospitals. CRBSIs were the source of a large proportion of the isolates (46%; *n* = 299). The majority of isolates (63.2%; *n* = 411) were *Staphylococcus epidermidis*; other CoNS species occurring relatively frequently included *Staphylococcus hominis* (12.6%; *n* = 82), *Staphylococcus haemolyticus* (9.8%; *n* = 64) and *Staphylococcus capitis* (7.2%; *n* = 47).

The ceftobiprole MIC_50_ and MIC_90_ values across all 650 isolates were 1 and 2 mg/L, respectively, and no isolate had a ceftobiprole MIC > 4 mg/L (Table 1). Therefore, based on the EUCAST PK/PD non-species-specific breakpoint for ceftobiprole (4 mg/L), resistance to ceftobiprole was not detected in any of the 650 CoNS isolates. Furthermore, the majority of isolates (91.6%; *n* = 596) had a ceftobiprole MIC of < 4 mg/L. Of the 54 isolates with a ceftobiprole MIC of 4 mg/L, all were classified as MR and 79.6% (*n* = 43) were *S. haemolyticus* (Table 1). The ceftobiprole MIC was ≤ 1 mg/L for all MS isolates.

**Table 1:** MIC distribution of ceftobiprole against CoNS isolated from bacteraemias at centres in the UK and Ireland in 2013–2015

Organism (no. tested)	No. of isolates (cumulative %) inhibited at MIC (mg/L) of									MIC (mg/L)	
	0.015	0.03	0.06	0.125	0.25	0.5	1	2	4	50%	90%
*S. capitis* (47)	2 (4.3)	7 (19.1)	7 (34.0)	8 (51.1)	2 (55.3)	9 (74.5)	10 (95.7)	2 (100)		0.125	1
*S. epidermidis* (411)			3 (0.7)	35 (9.2)	62 (24.3)	96 (47.7)	171 (89.3)	39 (98.9)	5 (100)	1	2
*S. haemolyticus* (64)				1 (1.6)	0 (1.6)	6 (10.9)	3 (15.6)	11 (32.8)	43 (100)	4	4
*S. hominis* (82)			1 (1.2)	6 (8.5)	17 (29.3)	13 (45.1)	12 (59.8)	28 (93.9)	5 (100)	1	2
*S. lugdunensis* (10)					1 (10.0)	8 (90.0)	0 (90.0)	1 (100)		0.5	0.5
*S. warneri* (20)				2 (10.0)	5 (35.0)	5 (60.0)	7 (95.0)	1 (100)		0.5	1
Other species (16)^a^			2 (12.5)	3 (31.3)	2 (43.8)	6 (81.3)	1 (87.5)	1 (93.8)	1 (100)	0.5	2
All isolates (650)	2 (0.3)	7 (1.4)	13 (3.4)	55 (11.8)	89 (25.5)	143 (47.5)	204 (78.9)	83 (91.7)	54 (100)	1	2

^a^Organisms include *Staphylococcus cohnii* (1), *Staphylococcus pasteuri* (3), *Staphylococcus pettenkoferi* (6), *Staphylococcus saprophyticus* (1), *Staphylococcus simulans* (4) and unspeciated *Staphylococcus* (1)

*CoNS* coagulase-negative staphylococci, *MIC* minimum inhibitory concentration

Among the 650 isolates, 471 (72.5%) were MR based on oxacillin MICs (Table 2). Methicillin resistance was common in all species where more than ten isolates were collected, with rates ranging from 46.8% in *S. capitis* to 96.9% in *S. haemolyticus*. Of the ten *S. lugdunensis* isolates, only one was MR. Across the entire collection, vancomycin resistance was only observed in a single isolate of *S. capitis* (Table 2). Overall, considerable resistance to ciprofloxacin (50.5%) and erythromycin (68.2%) was observed, while there was a lower level of resistance to clindamycin (25.1%) and teicoplanin (20.9%).

**Table 2:** Comparative in vitro activity of ceftobiprole against 650 CoNS from bacteraemias

Organism (*n*)	Antimicrobial agent	MIC determination			EUCAST MIC interpretation	
		MIC_50_ (mg/L)	MIC_90_ (mg/L)	MIC range (mg/L)	Percentage susceptible	Percentage resistant
All (650)	Ceftobiprole	1	2	0.015–4	100^a^	0.0^a^
	Oxacillin	16	> 128	≤ 0.03–> 128	27.5	72.5
	Ciprofloxacin	2	64	0.06–> 128	49.5	50.5
	Clindamycin	0.125	> 128	≤ 0.03–> 128	74.9	25.1
	Erythromycin	64	> 128	0.125–> 128	31.8	68.2
	Teicoplanin	4	8	0.25–> 16	79.1	20.9
	Vancomycin	2	4	0.5–8	99.8	0.2
*S. capitis* (47)	Ceftobiprole	0.125	1	0.015–2	100^a^	0.0^a^
	Oxacillin	0.25	> 128	0.06–> 128	53.2	46.8
	Ciprofloxacin	0.25	4	0.125–8	89.4	10.6
	Clindamycin	0.125	0.25	0.06–> 128	95.7	4.3
	Erythromycin	0.5	> 128	0.125–> 128	70.2	29.8
	Teicoplanin	1	8	0.5–> 16	89.4	10.6
	Vancomycin	2	2	1–8	97.9	2.1
*S. epidermidis* (411)	Ceftobiprole	1	2	0.06–4	100^a^	0.0^a^
	Oxacillin	8	128	≤ 0.03–> 128	24.6	75.4
	Ciprofloxacin	4	64	0.125–128	40.4	59.6
	Clindamycin	0.125	> 128	0.06–> 128	68.6	29.0
	Erythromycin	64	> 128	0.125–> 128	28.7	71.3
	Teicoplanin	4	8	0.5–16	73.2	26.8
	Vancomycin	2	4	1–4	100	0.0
Oxacillin-susceptible *S. epidermidis* (101)	Ceftobiprole	0.25	0.25	0.06–0.25	100^a^	0.0^a^
	Ciprofloxacin	0.25	8	0.125–128	88.1	11.9
	Clindamycin	0.125	0.125	0.06–> 128	92.1	7.9
	Erythromycin	0.5	> 128	0.125–> 128	52.5	47.5
	Teicoplanin	4	8	0.5–16	88.1	11.9
	Vancomycin	2	2	1–4	100	0.0
Oxacillin-resistant *S. epidermidis* (310)	Ceftobiprole	1	2	0.125–4	100^a^	0.0^a^
	Ciprofloxacin	8	64	0.125–128	24.8	75.2
	Clindamycin	0.125	> 128	0.06–> 128	61.0	35.8
	Erythromycin	> 128	> 128	0.125–> 128	21.0	79.0
	Teicoplanin	4	8	1–16	68.4	31.6
	Vancomycin	2	4	1–4	100	0.0
*S. haemolyticus* (64)	Ceftobiprole	4	4	0.125–4	100^a^	0.0^a^
	Oxacillin	> 128	> 128	0.125–> 128	3.1	96.9
	Ciprofloxacin	16	128	0.125–> 128	23.4	76.6
	Clindamycin	0.125	> 128	0.06–> 128	73.4	26.6
	Erythromycin	> 128	> 128	0.25–> 128	3.1	96.9
	Teicoplanin	4	8	1–> 16	78.1	21.9
	Vancomycin	2	4	1–4	100	0.0
*S. hominis* (82)	Ceftobiprole	1	2	0.06–4	100^a^	0.0^a^
	Oxacillin	8	128	0.06–> 128	35.4	64.6
	Ciprofloxacin	0.25	64	0.06–> 128	69.5	30.5
	Clindamycin	0.125	> 128	0.06–> 128	85.4	13.4
	Erythromycin	> 128	> 128	0.25–> 128	24.4	75.6
	Teicoplanin	0.5	4	0.25–8	91.5	8.5
	Vancomycin	1	2	0.5–4	100	0.0
*S. lugdunensis* (10)	Ceftobiprole	0.5	0.5	0.25–2	100^a^	0.0^a^
	Oxacillin	0.5	0.5	0.25–128	90.0	10.0
	Ciprofloxacin	0.25	0.25	0.125–2	90.0	10.0
	Clindamycin	0.06	0.125	0.06–0.125	100	0.0
	Erythromycin	0.125	0.25	0.125–> 128	90.0	10.0
	Teicoplanin	1	1	1–1	100	0.0
	Vancomycin	1	1	1–2	100	0.0
*S. warneri* (20)	Ceftobiprole	0.5	1	0.125–2	100^a^	0.0^a^
	Oxacillin	64	> 128	0.125–> 128	30.0	70.0
	Ciprofloxacin	0.5	0.5	0.25–64	95.0	5.0
	Clindamycin	0.06	0.125	0.06–> 128	95.0	5.0
	Erythromycin	0.25	> 128	0.125–> 128	70.0	30.0
	Teicoplanin	2	4	1–4	100	0.0
	Vancomycin	2	2	1–4	100	0.0
Other species (16)^b^	Ceftobiprole	0.5	2	0.06–4	100^a^	0.0^a^
	Oxacillin	0.5	> 128	≤ 0.03–> 128	43.8	56.2
	Ciprofloxacin	0.25	4	0.125–8	87.5	12.5
	Clindamycin	0.125	0.5	≤ 0.03–> 128	87.5	6.3
	Erythromycin	0.25	> 128	0.125–> 128	68.8	31.3
	Teicoplanin	2	4	0.5–4	100	0.0
	Vancomycin	2	2	1–2	100	0.0

^a^The non-species-specific PK/PD breakpoint was used to determine ceftobiprole resistance

^b^Organisms included *Staphylococcus cohnii* (1), *Staphylococcus pasteuri* (3), *Staphylococcus pettenkoferi* (6), *Staphylococcus saprophyticus* (1), *Staphylococcus simulans* (4) and unspeciated *Staphylococcus* (1)

*CoNS* coagulase-negative staphylococci, *EUCAST* the European Committee on Antimicrobial Susceptibility Testing, *MIC* minimum inhibitory concentration

Oxacillin-resistant *S. epidermidis* displayed high rates of resistance to erythromycin (79.0%) and ciprofloxacin (75.2%) and moderate rates of resistance to clindamycin (35.8%) and teicoplanin (31.6%). Oxacillin-susceptible *S. epidermidis* exhibited lower levels of cross-resistance to the other antibiotics tested compared with oxacillin-resistant *S. epidermidis*. The largest difference observed between the two groups was with ciprofloxacin; only 11.1% of the oxacillin-susceptible *S. epidermidis* isolates were resistant to ciprofloxacin, while 75.2% of the oxacillin-resistant *S. epidermidis* isolates were resistant. No *S. epidermidis* isolates were resistant to vancomycin, regardless of their susceptibility to oxacillin or teicoplanin.

## Discussion

This study investigated the in vitro activity of ceftobiprole and other relevant antimicrobial agents against recently isolated CoNS collected from patients with bacteraemia. Ceftobiprole demonstrated excellent in vitro activity across the different species, including against isolates with methicillin resistance.

The majority of cases of nosocomial bacteraemia are caused by CRBSIs, with CoNS being one of the most commonly involved pathogens [1, 12]. Indeed, in the current study, the underlying source of bacteraemia was a CRBSI in 46% of cases. CRBSIs have a significant impact on patients and healthcare systems, with mortality rates and duration of admission to intensive care units significantly increased in patients with a CRBSI, compared with uninfected patients [13].

Methicillin resistance is widespread among CoNS isolates [1]. Recent surveillance studies have found overall antibiotic resistance rates of approximately 80% for CoNS, with methicillin resistance particularly frequent in *S. haemolyticus* and *S. epidermidis* [14]. The findings presented here are consistent with these data, with 72.5% of the CoNS isolates found to be MR, and the highest methicillin resistance rates are observed for *S. haemolyticus* (96.9%) and *S. epidermidis* (75.4%). While methicillin resistance is generally reported to be significantly lower among most other CoNS strains, in this analysis, methicillin resistance rates for *S. capitis* and *S. warneri* were similar to that observed for *S. epidermidis*, suggesting that methicillin resistance may be increasing among these species.

*S. lugdunensis* has a unique microbiological and clinical profile compared to other CoNS species [1]. While commonly found as a skin commensal in healthy individuals, *S. lugdunensis* can cause an acute and highly aggressive form of infectious endocarditis, as well as abscesses and wound infections, urinary tract infections, and infections of intravascular catheters and other implanted medical devices [1]. For *S. lugdunensis*, methicillin resistance rates of 10.0% were observed in the present study, comparable with the 7.9% rate observed in a previous international surveillance study [1, 15].

In previous research, CoNS, and particularly MR CoNS, have demonstrated a high rate of resistance to multiple classes of antibiotics, including fluoroquinolones (ciprofloxacin), lincosamides (clindamycin) and macrolides (erythromycin) [16]. This is supported by the results presented here, where erythromycin and/or ciprofloxacin resistance was observed in all species, and clindamycin resistance was observed in all species apart from the clinically distinct *S. lugdunensis*.

As resistance to vancomycin is rare in CoNS isolates, and because the majority of isolates are MR, vancomycin is often the first-choice antimicrobial in the treatment of CoNS infections [1, 17]. The current findings are in agreement with the previous studies, with only 1 out of 460 isolates (an *S. capitis* isolate without methicillin resistance) displaying resistance to vancomycin. Vancomycin MIC creep, where increasing numbers of isolates are observed with high vancomycin MICs that nevertheless remain within the susceptible range, has been frequently reported in *S. aureus*, but has not yet been observed in CoNS [1]. However, the occurrence of vancomycin-intermediate heteroresistant *S. epidermidis* has been reported in the last 5 years in several locations [18]. In one such outbreak in a neonatal intensive care unit, several of the isolates collected were also non-susceptible to daptomycin [18]. Therefore, the potential for CoNS strains to develop reduced susceptibility to vancomycin should not be ignored.

This study found an overall rate of resistance of 20.9% for teicoplanin, with teicoplanin-resistant strains observed (in order of highest to lowest resistance) for *S. epidermidis*, *S. haemolyticus*, *S. capitis* and *S. hominis.* These results are highly comparable to those observed in a previous study of CoNS strains isolated from the UK and Ireland from 2001 to 2006, where an overall resistance rate of 20.8% to teicoplanin was observed [16]. The similarities between these studies suggest that teicoplanin resistance in CoNS species has not changed significantly in the UK and Ireland in more than a decade, and there is no evidence of cross-resistance between teicoplanin and vancomycin.

In general, resistance to all antibiotics except vancomycin was strongly associated with methicillin resistance. Among MR *S. epidermidis*, a particularly high rate of ciprofloxacin resistance was observed, and this rate was markedly higher than that observed in MS *S. epidermidis*. Previous studies have also observed high rates of ciprofloxacin resistance in MR CoNS species [16]. While there may be a molecular link between methicillin resistance and resistance to other antibiotics, it has been hypothesised that this cross-resistance is more likely due to the overall prevalence of antibiotic resistance among CoNS species in the nosocomial setting [19].

Rifampicin has recently been reintroduced as an adjunctive treatment to vancomycin for the treatment of CoNS infections; however, rifampicin data were not included in this comparison as rifampicin monotherapy is not advised for the treatment of CoNS infections due to the rapid emergence of spontaneous resistance [1, 20]. Furthermore, robust evidence demonstrating the efficacy of rifampicin against staphylococci is lacking, with a recent phase III study in adult patients with *S. aureus* infections failing to demonstrate clinical benefit from the addition of rifampicin to conventional antibiotic regimens [21].

The frequent occurrence of multidrug resistance in CoNS infections, together with the frequently chronic nature of the infections [1], represents a major clinical challenge. Novel antimicrobial agents with activity against multidrug-resistant CoNS are therefore required to more effectively treat these infections. Ceftobiprole, an advanced-generation cephalosporin, has previously demonstrated in vitro bactericidal activity against staphylococci, including against CoNS. In an in vitro time-kill model examining ceftobiprole activity against 6 CoNS strains, including 4 MR CoNS strains, there was at least a 3-log (99.9%) reduction in viable bacteria after 24-h exposure to a concentration of ceftobiprole equal to twice the MIC [8]. In this study, all 650 isolates were found to be susceptible to ceftobiprole; 91.7% were inhibited by a ceftobiprole concentration of 2 mg/L and the remainder inhibited by a ceftobiprole concentration of 4 mg/L, corresponding to the EUCAST PK/PD non-species-specific susceptibility breakpoint for ceftobiprole [11]. The three most common species observed in this analysis were *S. epidermidis*, *S. hominis* and *S. haemolyticus*, of which 98.9, 93.9 and 32.8% of isolates, respectively, were inhibited by ceftobiprole concentrations of 2 mg/L. The lower rate of inhibition in *S. haemolyticus* suggests that ceftobiprole may be less effective against infections caused by this strain, compared with *S. epidermidis* and *S. hominis.* Previous research has shown that *S. haemolyticus* may be particularly predisposed to acquiring antibiotic resistance, as its genome has high plasticity due to a high number of insertion sequences [22]. *S. haemolyticus* may thereby act as a reservoir for resistance genes that can be transferred to other staphylococci and therefore may be an important factor contributing to the development of antibiotic resistance in other CoNS species [22].

*S. capitis* was found to be the only species to demonstrate vancomycin resistance. *S. capitis* infection has been observed in neonates and adults with infective endocarditis or bone infections and has historically been viewed as susceptible to a broad range of antibiotics [1]. However, data have suggested the emergence of multidrug-resistant *S. capitis* in hospitals across Europe [23]. A recent study also identified *S. capitis* as a likely causative agent in nosocomial prosthetic joint infections, with multidrug-resistant *S. capitis* detected in approximately 28% of isolates [24]. *S. capitis* may be an emerging nosocomial pathogen and therefore further surveillance of this species is warranted. Ceftobiprole demonstrated activity against *S. capitis* in this study and should be investigated further as a potential treatment for these infections.

A further factor complicating the treatment of CoNS infections is the tendency of the causative bacteria to form biofilms on the surface of implanted or inserted devices [1]. Biofilm-associated bacteria often display greater resistance to antimicrobial therapy and frequently act as a source of reinfection, resulting in the need for removal and replacement of the device. Ceftobiprole has demonstrated in vitro activity against both MR and MS isolates in a biofilm model, which may be an important attribute for potential clinical use in CoNS-related infections [25].

A limitation of this analysis is that it does not report susceptibility data for linezolid, which is often used in the treatment of CoNS infections, as this antibacterial agent was not included in the testing panel for the period considered.

In conclusion, as antibiotic resistance continues to evolve in CoNS species, new antibacterial agents are required, preferably with differentiated mechanisms of action that have wide coverage of the different species in the CoNS group. Ceftobiprole exhibited in vitro activity against all CoNS species tested, including *S. capitis* and MR isolates, but may have somewhat lower activity against *S. haemolyticus* than against other CoNS species. Based on the in vitro activity observed, the collection of clinical data regarding the efficacy of ceftobiprole in CoNS infections is warranted.
